# Evaporative cooling of speleothem drip water

**DOI:** 10.1038/srep05162

**Published:** 2014-06-04

**Authors:** M. O. Cuthbert, G. C. Rau, M. S. Andersen, H. Roshan, H. Rutlidge, C. E. Marjo, M. Markowska, C. N. Jex, P. W. Graham, G. Mariethoz, R. I. Acworth, A. Baker

**Affiliations:** 1 Connected Waters Initiative Research Centre, UNSW Australia, 110 King Street, Manly Vale, NSW 2093, Australia; 2 Connected Waters Initiative Research Centre, UNSW Australia, Sydney, NSW 2052, Australia; 3 Affiliated to the National Centre for Groundwater Research and Training, Australia; 4 School of Geography, Earth and Environmental Sciences, University of Birmingham, Edgbaston, Birmingham, B15 2TT, UK; 5 Mark Wainwright Analytical Centre, UNSW Australia, Sydney, NSW 2052, Australia; 6 Australian Nuclear Science and Technology Organisation, Lucas Heights NSW 2234, Australia; 7 Water Research Centre, UNSW Australia, Sydney, NSW 2052, Australia

## Abstract

This study describes the first use of concurrent high-precision temperature and drip rate monitoring to explore what controls the temperature of speleothem forming drip water. Two contrasting sites, one with fast transient and one with slow constant dripping, in a temperate semi-arid location (Wellington, NSW, Australia), exhibit drip water temperatures which deviate significantly from the cave air temperature. We confirm the hypothesis that evaporative cooling is the dominant, but so far unattributed, control causing significant disequilibrium between drip water and host rock/air temperatures. The amount of cooling is dependent on the drip rate, relative humidity and ventilation. Our results have implications for the interpretation of temperature-sensitive, speleothem climate proxies such as δ^18^O, cave microecology and the use of heat as a tracer in karst. Understanding the processes controlling the temperature of speleothem-forming cave drip waters is vital for assessing the reliability of such deposits as archives of climate change.

Continuous measurements of cave drip water temperatures at the source have never before been reported in the literature, nor have the controls on their temperature been explored systematically. However, temperature is a fundamental biogeochemical variable of direct relevance to micro-ecological processes in cave systems, and many geochemical reactions are sensitive to variations in temperature^1^. For example, the δ^18^O of calcite, a widely used speleothem climate proxy, is subject to temperature dependent fractionation^2^. Thus, for assessing the reliability of such deposits as archives of climate change, understanding the processes controlling the temperature of speleothem-forming cave drip waters is of fundamental importance.

After rainwater has infiltrated into the subsurface, its temperature is altered during flow through soil and bedrock and when exfiltrating by various in-cave processes. Slow, porous-matrix flow is likely to result in drip waters in thermal equilibrium with the rock matrix. Thus at depths of more than a few meters it will normally reflect the average annual ground surface temperature. However, where fracture flow predominates, more variable drip water temperatures may result even at greater depths and a variable degree of thermal disequilibrium between the water and the surrounding material may occur. The degree of thermal disequilibrium will depend on the flow rate, fracture aperture and thermal properties of the fluid and rock. Once water has reached an air-filled cavern its temperature will then also be influenced by the ambient air temperature in the cave. The cave air temperature may itself be variable due to breathing or other ventilation effects^3^,^4^. Cooling or heating associated with latent heat changes during evaporation or condensation has also been hypothesized but never directly measured in caves^5^.

Determining the relative importance of these various processes is challenging; the characterization of hydrologic flow and heat transport processes in fractured rock environments may be very complex in the shallow subsurface where the combined influences of soil structure, weathering profiles and regolith development result in highly variable modes of shallow flow that in turn influence flow rates and water transit time to deeper parts of the groundwater system^6^,^7^. Recent improvements in the cost, miniaturization and accuracy of temperature logging devices have greatly advanced the understanding of thermal transport in porous media^8^,^9^,^10^,^11^. However, within fractured rocks, most studies using heat as a tracer are restricted to inferring active fracture flow within boreholes from temperature anomalies^12^. Despite recent work on mechanisms of heat exchange in karst conduits^13^,^14^, temperature controls have yet to be systematically explored within karst infiltration waters.

Our objectives are to test the relative importance of the processes that may affect speleothem drip water temperature by using an innovative approach combining the first use of high resolution temperature and drip rate monitoring in a cave. We test the hypothesis that when evaporation occurs, it causes significant cooling and therefore disequilibrium between air, water and rock temperatures. Two transient drip flow events were initiated over a shallow speleothem sequence (a flowstone draining on to a stalactite) by irrigating the land surface. The response of these artificial events was contrasted to a speleothem (stalactite) with a natural low constant drip rate deeper in the cave. We demonstrate that drip water temperature may be significantly out of equilibrium with host air and rock temperatures, and that this has implications for speleothem climate proxies, such as δ^18^O in limestone formations, cave micro-ecology and the use of heat as a tracer in karst systems.

## Results

### Site characterisation

The Cathedral Cave field site is part of the Wellington Caves Reserve (Latitude −32.622°, Longitude 148.940°) in New South Wales, Australia located within the temperate semi-arid zone with rainfall of approx. 620 mm/a and a significant seasonal temperature variation (approx. −5 to 45°C). Evapotranspiration greatly exceeds precipitation causing a soil moisture deficit for the majority of the year. Meteorological parameters are continuously monitored at the nearby Wellington Hill Weather Station (data downloaded from http://groundwater.anu.edu.au). The cave entrance, at 325.2 m elevation, is situated close to the top of a north-south trending ridge formed from Devonian Garra Formation limestone. The cave is overlain by degraded box grass woodland, with bare soil and sparse tree cover^15^. The cave has previously been studied for its characteristics of drip-water behavior^16^,^17^,^18^,^19^.

**Figure 1:**
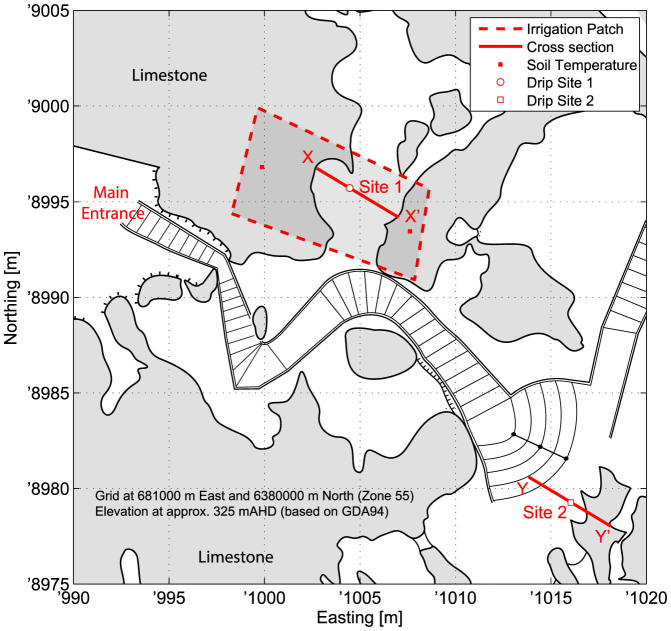
Site map (modified map courtesy of Sydney University Speleological Society).

Background observations of the cave climatology are consistent with it being a descending dead-end cave. Two entrances, located at the same elevation in the entrance series, could lead to limited ventilation in that part of the cave. Air exchange close to the entrance would also be expected through pressure and density effects. Air temperature measurements using Star-Oddi micro loggers show little variation in the deepest parts of the cave (18.25 ± 0.05°C, August 2011 to August 2012) over the long-term. Near the entrance (Site 1, Fig. 1), we have previously logged temperature variability over the short term and observed a range of 17–18°C in January 2013.

**Figure 2:**
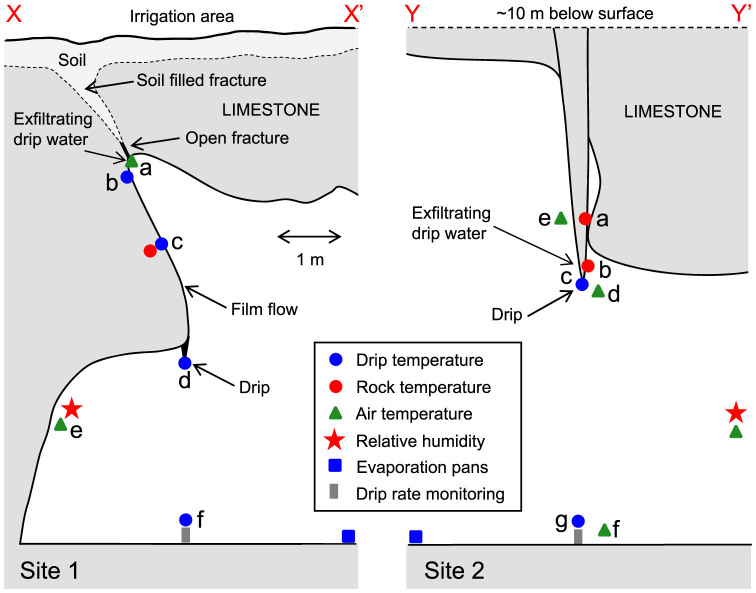
Vertical cross sections at Site 1 and 2 showing the location of the instrumentation.

Based on the site investigation, a shallow and heterogeneous soil covers a massively bedded limestone with variable topography. Below the soil, fractures create a hydrological connection between the irrigation area and the cave at Site 1 situated approximately 2 m below the ground surface (Figs. 1 and 2). The flow pathway connecting Site 2 to the surface is uncertain (the site is more than 10 m below the ground surface) but is likely connected to a relatively large water store in the karst formation above yielding a persistent and steady drip rate in comparison to the ‘flashy' nature of the Site 1 hydrology.

### Experimental results

**Figure 3:**
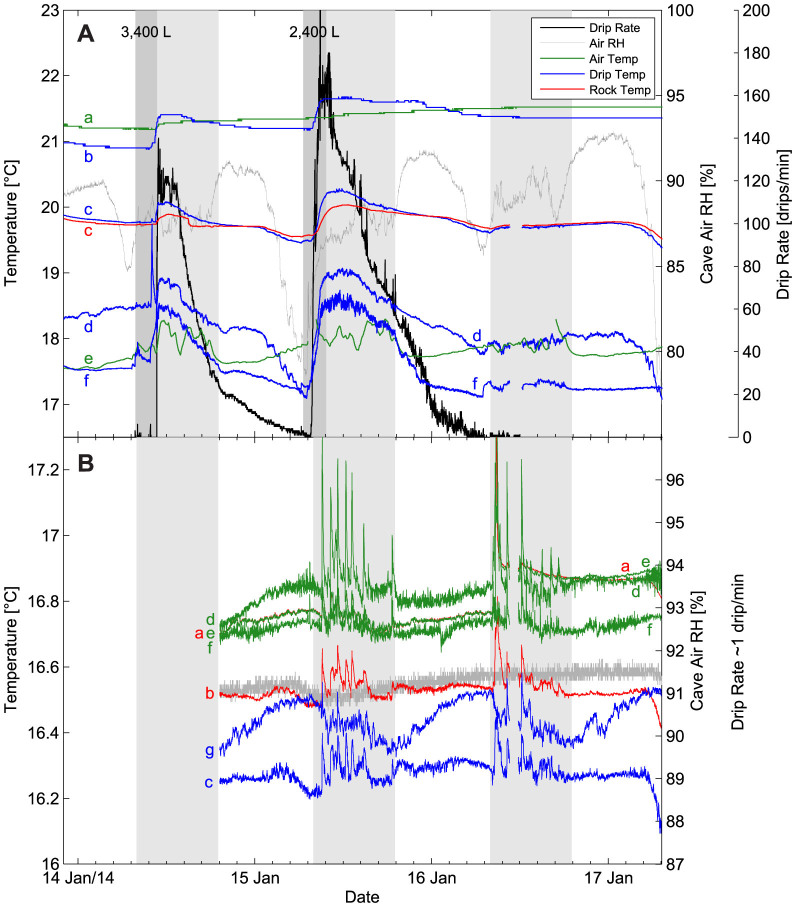
Observed soil, air, rock and drip water temperatures, drip rates and relative humidity for (A) Site 1 and (B) Site 2. Site 2 drip-rate was approximately 1 drip/min for the whole duration of monitoring. Periods of variable human impact on the temperature data are indicated by light grey bars, and the timing of the two irrigation applications are marked by dark grey bars.

The time series of temperatures, relative humidity and drip rates for Sites 1 and 2 are shown in Fig. 3. Periods of human activity in the cave (from ~8 am to ~ 7 pm denoted by light grey bands in Fig. 3) show a clear influence on the temperatures, and the data are therefore more variable during these periods. Because the cave is a show-cave with guided tours the influence of small numbers of people (~10–20) entering the cave at approximately hourly intervals can be seen clearly, especially at Site 2 which is closer to the tour route (see temperature spikes in Fig. 3).

At Site 1, under dry conditions prior to irrigation, a downward temperature gradient of approximately 1°Cm^−1^ was present from the exfiltration point to the drip point in both rock and air (Fig. 3A). This is consistent with observations of surface air and soil temperatures indicating that this shallow site is subject to seasonal temperature changes propagating from the surface by conduction, with strong downward warming at the time of the experiment (southern hemisphere mid-summer). This temperature gradient in the cave air was creating a self-sustaining stable vertical density distribution of the air-mass. Nearly identical temperatures within the rock (at 4 cm into the rock mass) and at the dry rock surface at Site 1c (Figs. 2 and 3A) suggest equilibrium between the rock and air prior to irrigation.

Following the onset of flow into the cave a similar spatio-temporal pattern of temperature responses was observed for both irrigations. Firstly, the exfiltrating water temperature increased above the ambient air temperature as heat was ‘washed in' by advection from the warmer rock mass above (Site 1b, Figs. 2 and 3A). This advective signal mirrors the shape of the drip flow hydrograph as measured by the Stalagmate drip logger. As the water flows down the flowstone, heat is then exchanged with the rock mass indicated by a lagged and attenuated temperature response at 4 cm depth at Site 1c (Figs. 2 and 3A) in comparison with the temperature of the water film. At the beginning of each flow event, the advective heat pulse is strong enough to be seen at the stalactite (Site 1d) and at the cave floor (Site 1f).

However, as the flow rate decreases, a second process controlling the drip temperatures becomes apparent – a strong cooling effect which increases in magnitude along the flow path until the point of drip formation on the stalactite. By comparing the relative air and drip water temperature changes at Sites 1d and 1e it is apparent that the cooling has a maximum amplitude of approximately 1.5°C (e.g. at 7 am on 15 Jan and 17 Jan). This effect is explained by latent heat loss due to evaporation driven by a relative humidity below 93% during the period of observations; the maximum observed cooling coincides with periods of lowest relative humidity. The evaporation rate was measured as an average of 0.14 ± 0.02 mm/d at the cave floor at Site 1 over the duration of the experiment. However, relative humidity was found to vary during a roving survey at the end of the experiment (17/1/2014 at 8:00) from 85% at the cave floor, to 95% adjacent to Site 1a indicating that the evaporation rate from the film of water on the flow path would have been significantly lower than the value measured by volumetric loss at the cave floor. The evaporative cooling is clear but less pronounced (maximum 0.8°C) on the Stalagmate drip logger (Site 1f, Figs. 2 and 3A) in comparison to the stalactite.

At Site 2, the ambient air temperature gradient was less than 0.05°Cm^−1^ between the stalactite and the cave floor, and no measurable temperature gradient was present between the air and dry rock mass at Site 2a, 2d or 2e (Figs. 2 and 3B), with temperatures at these sites only differing by a few hundredths of a °C. In contrast to Site 1, this is consistent with the Site's deeper position in the cave away from the zone of significant surface-driven seasonal temperature variations. The temperature of the probe mounted on the surface of the stalactite tip (Site 2c), over which the drip flowed, was persistently cooler than the ambient air temperature (Site 2d) by up to 0.7°C. The temperature probe mounted just above the stalactite tip, on the opposite side of the formation where it was always dry, was persistently cooler than the ambient air temperature by up to 0.4°C (Site 2b, Figs. 2 and 3B). These observations of cool drip water are again best explained by evaporation, with the cooling effect propagating through the stalactite and up the dry parts of the formation to Site 2b (Fig. 2). The site's position below approximately 10 m of rock mass combined with the very slow rate of flow preclude the possibility that this effect could otherwise be explained by the temperature of the water exfiltrating from the rock mass being significantly cooler than the rock mass itself. The temperature measured at the Stalagmate drip-logger (Site 2g, Figs. 2 and 3B) was also persistently cooler than the air temperature and varied diurnally. As for Site 1, a lower amount of cooling on the Stalagmate in comparison to the stalactite was observed. For Site 2 the evaporation measured by volumetric water loss was around 0.11 ± 0.02 mm/d with relative humidity being 91 to 92% during the period of observations.

Based on data from the nearby climate station, the drop in cave air relative humidity observed early in the morning (Fig. 3A) appears to be a result of the outside air density exceeding that of the in-cave air causing eddies and cave air ventilation at those times. This occurs despite the outside air temperature still being above that of the cave air because dry air is denser than moist air at the same temperature. The density driven ventilation also explains the small increase in air temperature observed during the night at both sites. Of the probes at Site 2, Site 2g, which is positioned at the base of steps leading from the cave entrance, shows the greatest warming effect. For Site 2c on the stalactite, the slight ventilation driven warming is more than offset by the increased cooling due to the drier and denser air increasing the evaporation rate. Given the high accuracy (0.001°C) of the temperature probes used, the apparent noise in the air temperature readings (for example, during the night when there is no human interference) in comparison to rock or water temperatures is also suggestive of smaller scale air circulation through density driven eddies.

While the number of existing studies which have actually measured or inferred cave evaporation in other caves is rather sparse, the literature (Table 1) shows clearly that the evaporation rates described in this paper sit within the range of values quoted in the literature observed in a variety of climates and deeper settings, some 100 s to 1000 s of meters from the cave entrances. As long as cave atmospheric water vapour content is low, or sufficient air exchange occurs^20^, conditions will be conducive to promoting evaporation, which will increase with decreased relative humidity or increased air flow^21^.

## Discussion

### Processes controlling speleothem temperatures

Novel irrigation experimentation and monitoring in a limestone cave has revealed new insights into three categories of processes controlling speleothem temperatures:

1. *Evaporative cooling*. Our observations support, for the first time, the hypothesis that evaporative cooling, in this case up to 1.5°C, can occur during film flow along a cave wall before reaching a drip point, even in a relatively high humidity environment (~90%). This may significantly affect the temperature of speleothem-forming water on stalactite drips and stalagmite caps as well as flowstone features. Although this type of film flow, and thus the potential for evaporative loss, was directly observable in our experiment, in other locations film flow within cavities with restricted access may not be observable. For such settings, an observed temperature difference between air and drip water could be used as an indication of this evaporative process higher in the cave system. The effect becomes the dominant control on drip temperature when cooling due to latent heat is greater than warming due to advective heat transport from the hydrologic system above the cave, or greater than heat transfer from or to cave walls or air. It is also notable that the evaporative cooling effect (up to 1.5°C) during the monitored period was greater that the variation in temperature caused by tourists (up to 0.5°C). Differences in magnitudes of cooling were observed between stalactite drips and water ponded on ground-based Stalagmate loggers. Further experimental work is underway to investigate the influence of the geometry, orientation, and thermal properties of a particular formation, and the water film thicknesses, on the relative cooling rate.
2. *Fracture flow and heat transport processes*. When drip-flow was initiated at Site 1, initial sharp increases in temperature in the drip water above the ambient cave air/rock temperature were caused by the rapid advection of heat through the lower, open part of the fracture bringing warmer (summer) water from higher in the profile down into the cave. During winter when the ambient temperature gradient is reversed we would expect this to reverse, e.g. result in an initially cold advective pulse of water entering the cave. This demonstrates that the subsurface hydrology can be an important control on drip water temperature in shallow systems.
3. *Cave air ventilation processes*. Although it has long been recognized that cave ventilation may affect cave air temperature^22^, we show here the importance this may have for cave drip water temperatures by also altering the cave air relative humidity which then controls the rate of evaporation and therefore the rate of cooling. In the present case, relative humidity changes in the cave are caused by ventilation initiated when the outside air density increases above that of the air at the entrance of the cave. This enables ongoing evaporative cooling due to air movement and a semi-continuous source of air with a lower relative humidity.

### Implications

Despite the increasing interest in the use of heat as a hydrological tracer, research has mainly focused on inferring hydraulic parameters using temperature data from borehole depth profiles^23^, hot spring discharge^24^, and near-surface sediments^11^. For the first time this study yields new insight into the complexity of surface and subsurface mechanisms controlling cave drip water temperatures. This includes the coupling of different heat transport processes and has direct relevance to applications of heat tracing within karst systems. For example, we have shown how relatively subtle changes in temperature due to advection from the overlying rock mass can be masked by cooling due to evaporation. This result would lead to spurious interpretations regarding heat transport without knowledge of the evaporative cooling process. The results also have implications for the understanding of cave microbiological and micro-ecological processes, since the local temperature regime around a speleothem feature is a fundamental control on microbial activity within, for example, biomineralisation processes^1^. Furthermore, variations in local temperature gradients may significantly influence the micro-ecological habitat patch distributions^25^. Evaporative cooling may thus increase the temporal and spatial dynamics of local temperature gradients in a way not previously imagined.

In the field of speleothem paleoclimatology, the δ^18^O of calcite is a widely used climate proxy over millennial and longer timescales applied to climatic regions spanning from monsoonal^26^ to semi-arid^27^. However, during calcite precipitation, δ^18^O is subject to a temperature dependent fractionation of −0.24‰ per °C^2^. Evaporative cooling is therefore a potential contributor to the variability of δ^18^O in speleothem records, alongside other known processes^28^,^29^, and is another factor that can contribute to increasing δ^18^O along growth layers in stalagmites (the “Hendy test”^30^). We observed a maximum evaporative cooling effect of 1.5°C which is equivalent to a δ^18^O variation of −0.36‰, (over three times greater than the analytical uncertainty of 0.1‰), and as large as the isotopic shifts expected due to natural and anthropogenic temperature variability in the Holocene and the last 100 years, respectively. We note that although we observe evaporative cooling of drip waters over the timescale of minutes to days, it is relevant to stalagmites deposited over longer time scales such as centuries to millennia. That is, for the periods when evaporative cooling occurs, then the effect is relevant for the same period of time that an associated speleothem is forming from the drip water. This might be a continuous evaporative cooling, which can be hypothesized to be experienced by a stalagmite fed by a slow, relatively constant drip, similar to our Site 2; or a discontinuous evaporative cooling, experienced by a speleothem fed by a more variable drip regime, similar to our Site 1.

Paleoclimatic proxy records have provided useful data to help constrain climate sensitivity for example with regard to the temperature response to an increase in anthropogenic greenhouse gas emissions^31^. When evaporative cooling of speleothems occurs, it will increase the uncertainty of such proxy paleo-temperature sensitivity estimates.

Other potential temperature proxies are contained within speleothems, such as the clumped isotope signature (*Δ*_47_)^32^ and indices derived from relative distributions of a class of lipid molecules glycerol dialkyl glycerol tetraethers (GDGTs), derived from microbial membranes^15^. Understanding the processes controlling the temperature of speleothem-forming cave drip waters is fundamentally important for assessing the reliability of such deposits as records of past climate change, especially with increasing research in both well-ventilated caves and cave entrances^20^,^33^,^34^, as well as caves in modern day or past semi-arid regions^35^,^36^,^37^. Furthermore in addition to the daily and sub-daily variations described in this study, the extent of evaporative cooling will likely also show seasonal and inter-annual variability. Such variability will be site specific, depending on drip rate amount and variability as well as cave climate parameters. Our results therefore have significant implications for the use of speleothems for paleoclimate reconstruction and the assessment of uncertainties regarding the likelihood of evaporative cooling being significant should be considered on a site specific basis. This could be achieved by simply comparing drip water and air temperatures using relatively inexpensive but well-calibrated temperature probes. We recommend that this should be routinely carried out (through a full seasonal cycle) at sites in which formations are being used for paleoclimate studies.

## Methods

Two sites (Site 1 and Site 2, Fig. 1) with contrasting hydrological regimes were instrumented and monitored during the period 13–17 January 2014, during the Australian summer, as follows.

Site 1 is near (~10 m) the cave entrance and, although it shows regular dripping due to natural precipitation events, was dry at the beginning of the experiment. After the site had been instrumented the ground surface above the site was irrigated twice to simulate two large rainfall events and initiate drip flow. The area was uniformly wetted by walking across the surface denoted in Figs. 1 and 2 with the outlet of two hoses attached to the mains water supply. The approximate volumes used were 3,400 L (on 14/01/2014 from 7:50 to 10:41, denoted by first dark grey band in Fig. 3A) and 2,400 L (on 15/01/2014 from 6:35 to 9:40, denoted by second dark grey band in Fig. 3A). This equates to 66 and 46 mm depth of simulated rainfall, respectively, and is typical of a large storm event. Previous experiments at the site (unpublished data) showed that drip temperatures in the cave were insensitive to applications of irrigation water of different temperatures (0 to 24°C). Hence, water direct from the town supply (26–30 C) was used without the need to control its temperature. Five drips were activated by the irrigation at Site 1, all flowing down a 3 m long flowstone feature after exfiltrating into the cave before dripping from stalactites (Fig. 2). Site 2 is deeper (~40 m) into the cave (Fig. 1) and at the time of the experiment a single drip from a 5 m long stalactite formation (Fig. 2) had a steady rate of approximately 1 drip/min which remained unaffected by the surface irrigation above Site 1. This drip emanated from the interior of the stalactite approximately 15 cm above the drip point, forming a film of water along one side of the formation. For logistical reasons no data were able to be collected from Site 2 on 14 Jan 2014.

The cave and the ground surface were surveyed to accurately map the geometry of each site with respect to the ground surface. A survey of soil depth was also carried out by probing with a metal rod. Each active drip at both sites was instrumented with drip counters (Stalagmate, Driptych, UK) aligned on the cave floor. The temperature of the water, rock and air was measured at various locations and depths into the rock mass along the flow paths (Fig. 2) using: (1) a PC based data acquisition system (USB-6225 DAQ, National Instruments, USA) in combination with a custom designed high-resolution (0.0006°C) multi-channel device to measure the resistance change of Platinum resistors (2 × 2 mm thin-film Pt1000; 3-wire connection per channel; sensors embedded in short sections of aluminum tube). Temperatures were calculated from the resistance change using the Callendar-Van Dusen relationship; (2) self-contained miniature temperature loggers (DST micro T, Star Oddi, Iceland). All temperature equipment was calibrated beforehand using a highly accurate temperature reference (Fluke, model 1524) resulting in probe accuracies of 0.001°C. Temperatures were measured at the rock surface using the flat 2 mm thick aluminum probes, fixed in place with a combination of small nails and adhesive, while ensuring minimal disturbance to the flow of water over the formations. Carbon-fiber rods with two temperature sensors spaced 40 mm apart were inserted 40 mm deep into 5 mm diameter holes drilled orthogonally to the rock surface. To ensure minimal intrusion of heat or water between rod and hole, the annular space was sealed with low thermal conductivity putty.

The air and soil temperature (at 8 cm depth) on the ground surface above the cave was also measured using Star Oddis. Relative humidity was measured adjacent to each drip site using Campbell Scientific data loggers and HMP155A probes. All measurements were recorded at 1 sample/minute. Glass evaporation pans (9.5 cm internal diameter) with initially 50 ml of water were placed, in duplicate, next to each site and the loss of water (due to evaporation) over the course of the experiment was measured volumetrically. The volume error on this estimate was ±0.5 ml.

## Author Contributions

Experimental design was carried out by M.O.C., G.C.R., M.S.A., H.R.u., C.E.M., A.B., P.W.G., H.R.o. & G.M. and fieldwork conducted by M.O.C., G.C.R., M.S.A., H.R.u., M.M., C.N.J. & A.B. Funding for the work was won by R.I.A. & A.B. M.O.C., G.C.R. & A.B. wrote the manuscript with input from all authors.

## Figures and Tables

**Table 1:** Review of literature values for cave evaporation. * denotes sites away from artificial lighting

Reference	Location, Climate	Evaporation rate (mm/a)	Distance from cave entrance (m)
Atkinson et al. (1983)^38^	Alberta Canada, continental/subarctic	14	1–1200
		1.4–2.5	1200–4200
De Freitas & Schmekal (2006)^39^	New Zealand, sub-temperate	0–21	≈60
Buechner (1999)^40^	US, arid	3–9	≈600
Mclean (1971)*^41^	US, semi-arid	120	≈250
		12	≈900
Current study	Australia, temperate semi-arid	40–50	10–40
